# Surgical Options for Initially Unresectable Colorectal Liver Metastases

**DOI:** 10.1155/2012/454026

**Published:** 2012-10-03

**Authors:** Irinel Popescu, Sorin Tiberiu Alexandrescu

**Affiliations:** Dan Setlacec Center of General Surgery and Liver Transplantation, Fundeni Clinical Institute, Carol Davila University of Medicine and Pharmacy, Fundeni Street No. 258, 022328 Bucharest, Romania

## Abstract

Although the frontiers of liver resection for colorectal liver metastases have broadened in recent decades, approximately 75% of these patients present with unresectable metastases at the time of their diagnosis. In the past, these patients underwent only palliative treatment, without the chance of a cure. In the previous two decades, several therapeutic strategies have been developed that render resectable those metastases that were initially unresectable, thus offering the chance of long-term survival and even a cure to these patients. The oncosurgical modalities that are available include liver resection following portal vein ligation/embolization, “two-stage” liver resection, one-stage ultrasonically guided liver resection, hepatectomy following conversion chemotherapy, and liver resection combined with thermal ablation. Moreover, in recent years, certain authors have recommended the revisiting of the concept of liver transplantation in highly selected patients with unresectable colorectal liver metastases and favorable prognostic factors. By employing such therapies, the number of patients with colorectal liver metastases who undergo a potentially curative treatment could increase to 40%. The safety profile of these approaches is acceptable (morbidity rates as high as 45%, mortality rates of less than 5%). Furthermore, the 5-year survival rates (approximately 30%) are significantly increased over those that were achieved with palliative treatment.

## 1. Introduction

The current treatment for patients with liver metastases from colorectal cancer is multimodal, including liver resection, chemotherapy, targeted therapies (monoclonal antibodies), interventional radiology, and radiotherapy. The complete resection of liver metastases results in 5-year overall survival rates that range from 21% to 58% [1–3], which are significantly higher than those rates that are achieved by nonsurgical therapies (5-year survival rates less than 5%) [4]. Thus, the only potentially curative therapy in patients with colorectal liver metastases (CRLM) includes complete resection of the liver metastases. 

At present, CRLMs are considered resectable when the following criteria are met [5, 6]:

- the complete resection of all known disease can be achieved,
- at least two contiguous liver segments can be preserved, with adequate vascular inflow and outflow, with biliary drainage,
- the remnant liver volume is adequate to avoid postoperative liver failure.

In patients with a healthy liver, the volume of the future liver remnant (FLR) should represent more than 25% of the total liver volume (TLV) to avoid postoperative liver failure [7–9]. However, in patients with chronic liver disease or chemotherapy-induced liver injury, a minimum of 40% of the TLV should be preserved [9–12].

Therefore, although the frontiers of liver resection have broadened over the previous two decades [13], approximately three quarters of patients with CRLM are not eligible for an initially curative liver resection (R0) after a preoperative evaluation [14].

The most common causes of the initial unresectability are the following.

1. A single, very large liver metastasis, the resection of which would not spare a sufficient volume of liver parenchyma to avoid postoperative liver failure.
2. Multiple bilobar liver metastases, the complete resection of which would not preserve a sufficient volume of functional liver parenchyma.
3. CRLM involving or located in close proximity to either the bifurcation of the portal vein or the confluence of the three hepatic veins with the inferior vena cava (IVC). In this case, the resection of the liver metastasis would not allow for the preservation of a minimum of two adjacent liver segments with adequate vascular inflow and outflow.

Until 20 years ago, the only available treatment for these patients was palliative chemotherapy, the goals of which were to increase progression-free and overall survival; however, there was no prospect of a cure. Although survival rates increased with the advent of new chemotherapeutics (such as Oxaliplatin and Irinotecan) and targeted therapies (e.g., Bevacizumab, Cetuximab, and Panitumumab), the current survival rates for these cases are still modest compared to those that can be achieved by liver resection. Therefore, several therapeutic strategies were introduced to achieve a complete resection in these patients [15].

## 2. Therapeutic Options

**Figure 1:**
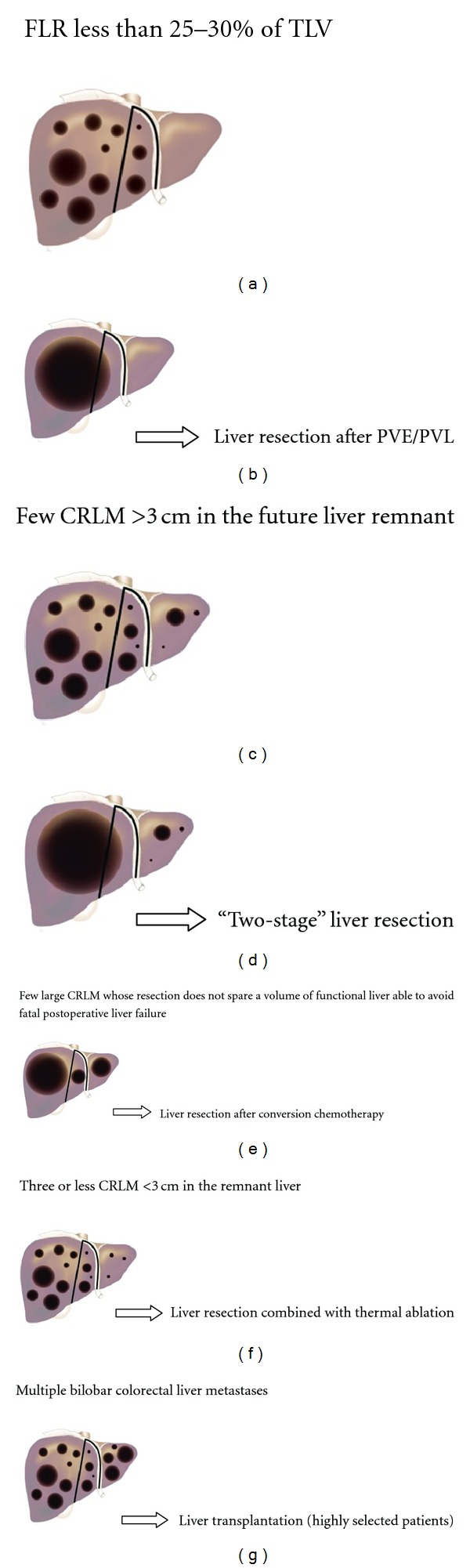
Strategies used for potentially curative treatment of the initially unresectable CRLM, depending on the location, number, and size of the lesions.

In Figure 1, we schematically present those situations in which metastases are considered unresectable and the therapeutic options that are available for conversion to resectability.

### 2.1. Liver Resection Following Portal Vein Embolization/Ligation

In certain instances, although a minimum of two adjacent segments with appropriate vascular inflow and outflow, and biliary drainage can be preserved following the complete resection of CRLM, the volume of the remaining liver parenchyma may be insufficient to avoid postoperative liver failure. Such situations are generally encountered in patients who (1) require a right trisectionectomy, or (2) when a right hemihepatectomy must be performed, but the volume of the left hemiliver is prohibitively small (Figures 1(a) and 1(b)). To avoid postoperative liver failure in these patients, it is advisable to attempt to increase the volume of the FLR prior to the liver resection. 

This goal may be achieved by initially performing portal vein embolization (PVE) or ligation (PVL). If the volume of the FLR following a PVE/PVL increases sufficiently to prevent the risk of postoperative liver failure, liver resection should be performed 4–8 weeks later.

This therapeutic strategy is based on the observation that increasing the volume of the FLR improves the function of the residual liver parenchyma following the hepatectomy [16, 17].

The reports of Kinoshita and Makuuchi revealed that the ligation or embolization of the right portal vein induces a process of atrophy-hypertrophy of the liver (increasing the safety of liver resection) in patients with hepatocellular carcinoma or hilar cholangiocarcinoma [18, 19]. Therefore, other authors applied the same procedure in patients with CRLM whose FLR was insufficient to avoid postoperative liver failure [10, 20–22]. The rationale for such an approach is that the embolization or ligation of the right portal branch abolishes the portal inflow into the right hemiliver, leading to its atrophy; alternatively, the portal inflow into the left hemiliver increases, causing hypertrophy of the FLR.

This approach permits the performance of the scheduled hepatectomy (while concomitantly reducing the risk of fatal liver failure) in more than 50% of patients who were otherwise unresectable due to a small FLR. 

Concerns regarding the comparative effectiveness of PVE versus PVL have been raised by certain authors. In an animal model, Furrer et al. revealed that the hypertrophy of the left hemiliver significantly increased following PVL versus PVE. These authors hypothesized that the entrapment of a greater number of macrophages in the embolized liver (due to the foreign-body reaction that is induced by the material used for embolization) explains this result [23]. Their conclusion that PVL is superior to PVE in inducing a regenerative response of the remnant liver is in contrast to that of Wilms et al., who stated that although PVL and PVE both induce liver hypertrophy, PVE is the most effective technique to increase the FLR [24]. These authors stated that PVE-induced vascular occlusion is more durable than that induced by PVL. Furthermore, the cause of the inferior regeneration in the ligation group was reported to be the formation of collaterals between the occluded and nonoccluded portions of the liver. To avoid this undesirable situation, certain authors recommended the transection and ethanol injection into the ligated portal branch [20]. Lastly, in addition to these experimental studies, a retrospective study of 35 patients revealed that PVL and PVE are similar in terms of both increasing the FLR and the conversion to resectability rate [25]. 

PVE- or PVL-induced liver hypertrophy involves both segments 2-3 and segment 4. Most patients that are subjected to PVL/PVE require a right trisectionectomy. In such patients, the principal objective of this maneuver is to increase the volume of segments 2-3 and not the volume of segment 4 (which is resected). To primarily increase the volume of the left lateral section, certain authors state that the optimum approach is to concomitantly occlude the right portal vein and the portal branches to segment 4 (right trisection portal vein embolization-R3PE) [26]. In 2000, Nagino et al. presented results that support this hypothesis, demonstrating that the volume gain of the left lateral section was higher in patients with R3PE relative to patients who received right portal vein embolization [26]. To date, we have performed R3PL in one patient, and the results were outstanding: the percent of FLR gain was 16.22%, whereas this metric was 10.8% in the patients who received a right portal branch ligation [27]. However, another study that was published in 2005 failed to confirm these results, revealing that the mean volume of segments 2-3 following embolization and the rate of the segments 2-3 volume increase were similar between the patients who received a R3PE and those that received a standard right portal vein embolization [7]. Therefore, definitive conclusions cannot be drawn regarding the usefulness of the embolization of the portal branches to segment 4, and further studies are required to clarify this subject. Most centers now prefer to routinely perform only right portal branch embolization/ligation in such patients.

To achieve a more marked and rapid hypertrophy of the FLR following portal vein ligation, Schnitzbauer et al. [28] and de Santibanes et al. [29] recently recommended the association of right portal vein ligation with “in situ liver transection/splitting”. Using this approach, the authors achieved a significant and more rapid hypertrophy of the FLR, enabling subsequent curative liver resection during the same hospitalization. The authors concluded that this technique induces the rapid and more robust growth of the FLR [28] than is reported with portal vein occlusion alone. Moreover, this approach allows for the performance of a staged liver resection during a single hospital stay [29]. 

 Two formulas can be used to calculate the percent of the FLR gain following PVE/PVL:

- (Volume of the FLR following PVE − Volume of the FLR prior to PVE) × 100/Volume of the FLR prior to PVE [30],
- %FLR following PVE − %FLR prior to PVE [12].

In a series of 30 patients, the percent of the FLR gain (calculated using the first formula) was 42% [10], whereas the FLR gain ranged from 9.7% to 13% in different series using the second formula [21, 27, 30]. In most patients, these percentages are generally sufficient to allow for a safe resection of liver metastases. 

When PVE is planned, Bevacizumab should be used with caution given that Aussilhou et al. revealed the detrimental effect of this medication on the FLR gain; this effect is especially strong in patients who are older than 60 years and received more than six cycles [31]. However, other authors have demonstrated that this monoclonal antibody does not impair liver regeneration following PVE [32].

The most severe complications of right PVE are liver hematoma, liver abscess, thrombosis of the left portal vein, portal hypertension, and cholangitis. In a meta-analysis that was performed by Aboulkhir et al., the morbidity rate following PVE was 2.2%, and the mortality was zero [30].

The resectability rate following PVE/PVL in different centers ranges from 60% to 88% [10, 21, 22]. The primary reason of failure to perform the curative hepatectomy is not insufficient hypertrophy of the FLR but rather the progression of the disease. In our series, 5 of 13 patients (38%) exhibited disease progression following PVL, precluding a curative liver resection. The other 8 patients underwent successful complete resection of the initially unresectable CRLM. The resectability rate was therefore 62% [27].

The morbidity and mortality rates that were observed following curative hepatectomy were less than 35% and 4%, respectively, in most series [10, 22]. 

The 5-year survival rate of these patients was approximately 38% [10, 21]. 

It must be noted that in patients with liver metastases in the FLR, this approach is not recommended, due to the risk of rapid growth of these metastases. Such approach may jeopardize the chances of a subsequent potentially curative liver resection. Such patients should undergo a “two-stage” liver resection (see below) to clear the remnant liver prior to the PVE [33].

### 2.2. Two-Stage Liver Resection

The term “two-stage” liver resection has been used by a small number of authors to define a strategy that consists of a single liver resection that is performed following PVL and which does not include a sequential liver resection [20]. Herein, we shall use the nomenclature of “two-stage” liver resection for those procedures that consist of two consecutive hepatectomies.

This therapeutic strategy is used in patients with multiple bilobar CRLM, whose resection will not spare a sufficient amount of liver parenchyma to avoid postoperative liver failure. These patients usually require a right hepatectomy or a right trisectionectomy along with wedge resections of the metastases that are located in the left hemiliver or in the left lateral section (Figures 1(c) and 1(d)).

To avoid such extensive resections, which are accompanied by a high risk of postoperative fatal liver failure, it is recommended that a complete resection of the liver metastases be achieved in a two-stage surgical procedure. In the first stage, a limited resection of the metastases from the left hemiliver or the left lateral section (future liver remnant) is performed. In stage two (following the regeneration of the FLR), the bulk of the metastatic burden is resected by a right hepatectomy or trisectionectomy. FLR regeneration is essential to minimize the risks of hepatic failure following the second operation. Thus, PVE/PVL may be suitable in patients with small FLR to increase the safety of the second hepatectomy. To facilitate the second operation and to avoid disease progression between the first and the second intervention, it may be useful to deliver systemic or locoregional chemotherapy to shrink the metastatic bulk. To minimize the inhibitory effects of the chemotherapeutic drugs on liver regeneration, the chemotherapy should be begun three weeks following the first hepatectomy. This sequence is necessary, as liver regeneration is essential to the feasibility of the second resection [34].

Such a therapeutic approach is especially useful in patients with synchronous bilobar CRLM [35] given that (1) it avoids the cumulative risks of a simultaneous primary tumor resection and major hepatectomy, and (2) it allows for the evaluation of the chemosensitivity of the liver metastases and the guiding of the adjuvant therapy following the second operation. The resection of the primary tumor is performed in the first stage, along with a limited resection of the metastases from the future liver remnant (generally from the left hemiliver or the left lateral section). Occasionally, a right portal vein ligation is also performed during the first operation. Short-course chemotherapy (systemic or loco-regional) should begin three weeks later. If the residual lesions will be stable or responsive to chemotherapy, the second liver resection should be performed. 

The results of such an approach were first published by the Paul Brousse group in 2000. This group reported a resectability rate of 81% [34]. In other series, the resectability rate ranged from 66% to 75% [27, 33].

Among the published series, the morbidity rates following the first resection were less than 31%, and the mortality rates were zero [33, 34].

The morbidity rate following the second liver resection ranges from 45% to 56% [27, 33, 34]. Despite these relative high morbidity rates, the mortality was zero in most series [27, 33]. Nonetheless, a mortality rate of 15% following the second operation was reported by Adam et al. [34]. The authors explained that this result was a consequence of the combination of (1) the diminished tolerance of such patients to perioperative complications due to their advanced neoplastic disease and (2) the effects of the adjuvant procedures that were used to facilitate liver resection (chemotherapy, PVE).

The 3-year survival rates of these patients ranged from 35% to 54% [27, 33, 34], with a median survival of 44 months from the diagnosis of liver metastases [34].

In selected patients with multiple bilobar colorectal liver metastases, a "two-stage" liver resection could be avoided, by performing ultrasonically-guided hepatectomy.

### 2.3. One-Stage Ultrasonically Guided Liver Resection

The implementation of ultrasonography in liver surgery dramatically alters the approach to liver metastases, permitting a more accurate diagnosis and challenging the traditional paradigms of liver resection. 

Intraoperative ultrasound (IOUS) allows for the detection of additional CRLM that were not revealed by preoperative imaging methods and is the most accurate technique for detecting liver tumors [36, 37]. However, standard IOUS may miss lesions that are smaller than 1 cm, especially in patients who are undergoing preoperative chemotherapy, whose CRLM exhibit a similar echo-pattern to that of the surrounding liver parenchyma. The use of contrast-enhanced IOUS (CE-IOUS) was demonstrated to improve the detection of CRLM and is the most sensitive and specific method for the diagnosis of CRLM [38].

In the mid 1980s, IOUS was first used to guide the puncture and balloon occlusion of the portal branch that feeds the portion of the liver to be resected, allowing for limited anatomical liver resections instead of major hepatectomies [39–41]. This technique decreased the risk of postoperative liver failure and was recommended principally in patients who have HCCs on liver cirrhosis.

In addition, IOUS offers a better estimation of the spatial relationships between the liver tumors and the intrahepatic vessels, permitting the resection of liver masses with the preservation of intrahepatic vascular structures even when the tumors are located in close proximity to major intrahepatic vessels. Furthermore, even when major hepatic vein(s) must be resected, color Doppler IOUS findings provide reliable information that may lead to the preservation of a portion of the liver parenchyma that is drained by those vein(s), avoiding major hepatectomies [42–45]. Thus, a novel liver resection technique was developed in recent years that is referred to as “ultrasonically guided hepatectomy”. This technique opened the door to new procedures that allow for radical but conservative liver resections, reducing the requirement for major hepatic resections [46–48].

Because many patients exhibit colorectal liver metastases that are considered unresectable due to the insufficient remnant liver parenchyma following major hepatectomies, the use of this surgical technique (which spares a significant amount of functional liver parenchyma) allows for the complete resection of the metastases, reducing the risk of developing postoperative liver failure. Thus, this technique was used more frequently in patients with CRLM.

Moreover, ultrasonically guided liver resection decreases the requirement for major hepatectomies, obviating the requirement for portal vein occlusion prior to the liver resection and/or the necessity of a “two-stage” liver resection in selected patients.

In patients with CRLM that are located in close proximity to major hepatic veins or near the first-degree portal branches, a major hepatectomy is still the main surgical option at most centers. If the remnant liver volume following a major hepatectomy is critically small, a liver resection following portal vein occlusion, either by PVE or PVL, is generally recommended. In such instances, the patient is exposed to an interventional radiology procedure or a laparotomy prior to the curative liver resection. Each of these procedures presents additional risks of morbidity [30]. The development of the ultrasonically guided hepatectomy in recent years permits a more limited liver resection of poorly located CRLM, avoiding the necessity of a prehepatectomy PVE/PVL. In a series of 22 patients who presented poorly located liver tumors and who were scheduled for initial ultrasonically guided liver resection, a limited resection with or without hepatic vein preservation was achieved in 91% cases, providing lower morbidity rates than major resections following PVE and no mortality [43]. The rate of local recurrence (at the transection surface) was zero at a mean follow-up period of 23 months. Because this approach avoids portal vein occlusion (and its associated morbidity), the comfort of the patient is also improved. Moreover, the resectability rate following portal vein occlusion does not exceed 60–88%, due to either insufficient hypertrophy of the remnant liver or disease progression in the interval between the portal vein occlusion and the liver regeneration [21, 22, 49]. When an initial ultrasonically guided hepatectomy is performed, the risk of disease progression is avoided, and the hypertrophy of the FLR is no longer necessary. Thus, the resectability rate that is achieved by the ultrasonically guided approach appears to be higher than those that are achieved by PVE/PVL, broadening the indications for curative surgery in cases of CRLM [43]. 

In patients with multiple bilobar CRLM, the ultrasonically guided technique may also represent an effective alternative to the “two-stage” hepatectomy, permitting a curative and conservative liver resection [44]. The advantages of this approach over the “two-stage” liver resection are the comfort of the patient, a lower morbidity rate [44], and an increased possibility of repeat resections if the patient develops recurrent metastases [50–52]. Furthermore, the recurrence rate following one-stage ultrasonically guided liver resection was similar to that reported after “two-stage liver resection”.

Due to the aforementioned benefits, the one-stage ultrasonically guided liver resection should be part of the armamentarium of the liver surgeon, especially in the context of patients with complex tumoral presentations.

### 2.4. Liver Resection Following Conversion Chemotherapy

This therapeutic strategy was first presented by the Paul Brousse group in 1996 [53] and is recommended in patients with a small number of large CRLM, the resection of which would not spare a sufficient amount of functional liver to prevent postoperative liver failure (Figure 1(e)). The goal of this approach is to “downsize” the liver metastases to an extent that allows for their complete resection. Therefore, a chance of a potentially curative liver resection is available to patients who otherwise may have only benefited from palliative treatment.

Until 20 years ago, the only efficient chemotherapeutic regimen that was used in patients with unresectable CRLM consisted of 5-Fluorouracil (5-FU) and Folinic acid. Although this chemotherapy increases the overall survival rates and the progression-free survival rates of these patients, the response rates were less than 23%, and only anecdotal cases of liver metastases that shrink sufficiently to allow for a subsequent curative hepatectomy were reported [54–56].

The advent of new chemotherapeutic agents such as Oxaliplatin and Irinotecan led to significantly better results. The response rates that have been achieved by FOLFOX and FOLFIRI regimens range from 40% to 56% [57–59]. A strong correlation was observed between the response rates and the resection rates of patients with initially unresectable CLRM [60]. Therefore, more patients became resectable following so-called conversion chemotherapy. Folprecht et al. thus concluded that resectability should be considered a new endpoint for preoperative chemotherapy, focusing on the curative potential of this oncosurgical treatment [60]. 

The Paul-Brousse group published their updated results in 2004, reporting a 12.5% rate of conversion to resectability in 1104 patients with initially unresectable CRLM (following an average of 10 courses of chemotherapy) [14]. Apart from these very large series of patients, other centers have subsequently reported similar results in smaller numbers of selected patients who presented with initially unresectable CRLM that were rendered resectable by different chemotherapy regimens [61–63]. 

In many reports, the morbidity rates following hepatectomy in patients with initially unresectable CRLM [14, 62, 64] ranges from 23% to 28%, which are similar to those rates that are observed in patients with initially resectable CRLM. However, certain authors have reported significantly higher incidences of postoperative complications in patients who receive resections following “downsizing” chemotherapy, raising concerns regarding the deleterious effects of the preoperative chemotherapy on the liver parenchyma (see below) [65, 66]. The postoperative mortality rates are reported to be less than 2% in most centers, which are similar to those rates that have been achieved in patients who did not receive preoperative chemotherapy [14, 53, 62, 66].

The 5-year survival rate of patients who were rendered resectable by chemotherapy was 33% in the Paul Brousse group, a rate that was higher than those that were achieved by new palliative chemotherapeutic regimens in similar patients [14]. Although this survival rate is significantly lower than what can be achieved in patients with initially resectable CRLM (*P* value = 0.01), the 5-year disease-free survival rate of 22% that was reported in initially unresectable patients appears to fully justify the efforts to render to resectability, these patients with otherwise dismal prognosis [14].

The addition of targeted therapies (e.g., Bevacizumab, Cetuximab, and Panitumumab) to chemotherapy regimens may be useful in further increasing the rate of conversion to resectability in initially unresectable lesions. This hypothesis was confirmed in a series of patients whose liver metastases were refractory to previous rounds of conventional chemotherapy. The advent of Cetuximab to the next-line chemotherapy rendered 7% of these patients resectable, with morbidity and mortality rates of 50% and 3.7%, respectively, and a median survival of 20 months [67]. This study, similar to those of Zorzi et al., demonstrated that monoclonal antibodies in combination with conventional chemotherapy have no detrimental effects on the safety of liver resection [32]. Furthermore, Gruenberger et al. revealed that Bevacizumab has little detrimental impact on liver regeneration following hepatectomy [68]. However, it should be noted that the use of vascular endothelial growth factor inhibitors (e.g., Bevacizumab) prior to major surgery increases the risks of bleeding and wound healing complications [69]. This therapy should be discontinued 5–8 weeks prior to the surgical intervention [68, 70]. 

Several issues should be kept in mind when deciding to take this therapeutic approach.

(i) The response to chemotherapy cannot be assumed to persist. Metastases occasionally shrink and become resectable following several cycles of chemotherapy. However, if the chemotherapy is continued, the metastases may regrow and again become unresectable, closing the “window of opportunity” for a potentially curative hepatectomy [71]. Therefore, if the systemic disease is controlled, the liver resection should be scheduled as soon as the metastases become resectable.

(ii) If the chemotherapy is continued beyond the point when the metastases become resectable, it is possible the liver metastases will become smaller and will no longer be visible on imaging (CT/MR/PET scans). Such metastases are referred to as “vanishing metastases.” Unfortunately, this “radiological complete response” or “clinical complete response” [72] does not indicate a cure, as a “pathologic complete response” is achieved in fewer than 20% of cases [73, 74]. In one-third of patients with radiological complete response, a laparotomy may reveal small metastases that were missed by the imaging methods or residual scars, the resection of which would reveal viable tumor cells. Alternatively, in patients without macroscopic residual tissue (on laparotomy) and negative (contrast-enhanced) intraoperative ultrasound, pathologic examination of the resected specimens, including liver segments where the metastases where initially located, revealed viable metastatic cells in 75% of cases [74, 75]. An indirect confirmation of the presence of viable tumor cells at the sites of the former metastases (which were invisible on laparotomy) is given by certain reports. In a series of patients with 31 CRLM that disappeared following chemotherapy and which were not observed on laparotomy, the resection of the initial metastases site was not performed. After a one-year follow-up, 23 (74%) metastases recurred in situ [74]. 

The survival benefit that is achieved by performing liver resection in patients with clinical complete response following chemotherapy was revealed by another study. Fourteen patients with radiological complete response following chemotherapy were not subjected to a liver resection, achieving a 5-year overall survival of 14% and a median survival of 30 months. In 25 patients who suffered from initially unresectable CRLM that were rendered to resectability by a FOLFOXIRI regimen and further resected, the 5-year survival rate was 43%, and the median survival was significantly higher (61 months, *P* value = 0.006) [62].

For these reasons, laparotomy is mandatory in patients with vanishing metastases, with the aim of resecting the macroscopic residual metastatic tissue or the sites of the initial CRLM (“blind resection”). 

The resection of the metastases sites is a very demanding operation, especially in patients with (initially) multiple metastases located deep in the liver. In such cases, computer-based virtual surgery planning is very useful, merging pre- and postchemotherapy computed tomography data. Recently, Oldhafer et al. presented such a surgical approach. Information that is processed using a computer is then intraoperatively transferred to the liver surface using an image-guided stereotactically navigated ultrasound dissector, enabling the surgeon to perform the resection [75]. 

In patients with initially multiple bilobar CRLM that become “invisible” following chemotherapy and that cannot be identified intraoperatively, it is frequently impossible to assume a complete resection of the metastatic sites. Thus, a small number of these “missing metastases” will remain. In such situations, Elias et al. recommended the placement of a chemotherapy catheter in the hepatic artery, allowing for a hepatic arterial infusion (HAI) with Oxaliplatin, along with systemic 5-FU and Folinic acid [76]. Using this approach, following a median follow-up period of 51 months, the missing metastases did not recur in 62% of the patients. The recurrence rate following HAI with Oxaliplatin was significantly lower than those that were noted in patients who were treated by systemic chemotherapy alone (*P* value = 0.01) [76]. 

Alternatively, it should be noted that a small number (4–15%) of CRLM that were treated by systemic chemotherapy prior to the liver resection achieved a complete pathologic response [72, 73, 77]. The complete pathologic response was observed both in patients with or without a radiologic complete response. The predictive factors for a complete pathologic response were age less than 60 years, maximum metastasis diameter of less than 3 cm, CEA levels at diagnosis below 30 ng/mL, an objective response following chemotherapy [72] and the use of hepatic arterial infusion chemotherapy [76]. The addition of Bevacizumab to the Oxaliplatin-based chemotherapeutic regimens did not appear to increase the incidence of the complete pathologic response (11.3% versus 11.6%; *P* value = 0.59) [78]. Patients with complete pathologic response achieved uncommonly high survival rates (76% at 5 years) [72].

(iii) Treatment with new chemotherapeutic drugs induces alterations of the nontumoral liver parenchyma, potentially impacting the results of the liver resection. 

The initial belief was that use of Irinotecan may cause nonalcoholic fatty liver disease (NAFLD), which represents a spectrum of diseases. The mildest form of NAFLD is macrovesicular steatosis, and the most severe form is non-alcoholic steatohepatitis (NASH) [79]. Although a study that was published in 2003 revealed a correlation between prior treatment with chemotherapy and steatosis and highlighted the fact that morbidity rates following liver resections were significantly higher in patients with marked steatosis [80], more recent studies have reported differing results. In 2006, a multicenter trial revealed that Irinotecan was associated with steatohepatitis but not with steatosis [81]. Moreover, this latter study noted that the mortality rate was significantly higher in patients with steatohepatitis (14.7%) than in patients without steatohepatitis (1.6%, *P* value = 0.001).

The first study to reveal a correlation between Oxaliplatin and non-tumoral liver parenchyma injury was published in 2004 [82]. The results indicated that 78% of the patients who were preoperatively treated with Oxaliplatin exhibited sinusoidal alterations. These results were subsequently confirmed by other reports [83–85], which revealed that Oxaliplatin-based preoperative chemotherapy was associated with sinusoidal dilatation and congestion, peliosis, and venoocclusive disease. One of these studies reported that only long-course Oxaliplatin-based chemotherapy (6 or more cycles) is significantly associated with sinusoidal injury [85]. However, in a series that was presented by Vauthey et al., the risk of sinusoidal dilatation did not appear to increase with the duration of chemotherapy (although their patients received relatively short-course treatments) [81]. Interestingly, the addition of Bevacizumab to Oxaliplatin-based regimens appears to reduce the incidence and severity of hepatic injury [78]. The impact of these liver injuries on the clinical outcome of the patients who received resections following Oxaliplatin-based chemotherapy was assessed in several reports. None of these studies reported increased mortality rates following liver resection in patients with Oxaliplatin-related sinusoidal injury [79]. Two trials revealed that a limited course (fewer than 6 cycles) of Oxaliplatin-based therapy was not associated with increased morbidity rates following liver resection [81, 86]. However, Karoui et al. observed a statistically higher incidence of postoperative complications in patients who underwent a major hepatectomy following preoperative chemotherapy when compared with the patients who were subjected to a similar liver resection without preoperative chemotherapy [66]. Similarly, Nakano et al. observed that sinusoidal injury was significantly associated with increased morbidity and longer hospital stays in patients who underwent a major hepatectomy [85]. 

 The above-mentioned pitfalls that are associated with preoperative chemotherapy justify the scheduling of the liver resection as soon as the metastases become resectable.

### 2.5. Liver Resection Combined with Thermal Ablation for Unresectable CRLM

This type of approach is especially recommended in patients with multiple bilobar CRLM who present with fewer than 3 liver metastases in the FLR, with each of these metastases being less than 3 cm in maximum diameter (Figure 1(f)). Another indication for this approach is when one or a small number of metastases are anatomically poorly located (e.g., in close proximity to the confluence of the three hepatic veins and the inferior vena cava or at the bifurcation of the portal vein) [87].

 The operation consists of the resection of the main tumor bulk (generally by a right trisectionectomy) and thermal ablation of the unresected metastases from the remnant liver (frequently the left lateral section) [88].

This approach could be performed in a “two-stage” manner in patients with synchronous unresectable CRLM, as described by Lygidakis et al. [89]. In the first stage, the following procedures are performed: (1) the resection of the primary colorectal tumor, (2) the ligation and transection of the relevant (right or left) primary portal branch, (3) the ablation of the metastatic nodules in the contralateral hemiliver, and (4) the insertion of an arterial catheter into the hepatic artery for locoregional chemo(immuno)therapy. The second stage of the operation consists of the resection of the tumoral liver (usually by right hemihepatectomy or trisectionectomy). 

 Thermal ablation can be achieved using radiofrequencies, microwaves, lasers, or cryotherapy. 

To increase the chances of a complete hyperthermic ablation, the Pringle maneuver can be performed during the ablation [88].

The morbidity and mortality rates (44% and 2.3%, resp.) [27, 90] of patients undergoing combined liver resection and thermal ablation of unresectable CRLM appear to be similar to those of patients with initially unresectable CRLM that are rendered resectable by other therapeutic strategies. 

 A retrospective study that was published in 2004 reported a significantly higher local recurrence rate following resection combined with RFA (5%) than was observed following complete resection of the CRLM (2%). However, the patients were not stratified according to the maximum diameter of the ablated CRLM in this study [90]. It was recently demonstrated that the best results achieved by thermoablation are observed in patients whose CRLM were less than 3 cm in maximum diameter. Thus, a significantly higher rate (*P* value = 0.0001) of sustained complete ablation was achieved in patients whose lesions were less than 3 cm (66.7%) than for the patients with metastases that were larger than 3 cm (33.3%) [91]. One retrospective study (including resectable and unresectable CRLM) revealed that the local recurrence rate following the radiofrequency ablation (RFA) of metastases that were less than 3 cm in maximum diameter was 1.3% after a follow-up period of 33 months [92]. Moreover, another retrospective study (including patients with single CRLM treated by RFA or liver resection) determined that the 5-year overall and local recurrence-free survival rates were similar for patients with CRLM that were smaller than 3 cm who were treated either with RFA or liver resection [87]. 

 However, in patients with multiple bilobar CRLM who are treated with a combination of RFA and liver resection, the overall and disease-free survival rates were significantly lower than for patients who underwent a complete resection of CRLM but significantly higher than in the patients who were treated by chemotherapy alone [90]. Moreover, Rivoire et al. revealed in a study of 57 patients with initially unresectable CRLM that the overall survival rates were similar for patients who underwent a complete liver resection following conversion chemotherapy and those who received liver resection combined with cryotherapy [93].

 These results justify liver resection combined with thermal ablation of initially unresectable CRLM in patients who fulfill the above-mentioned criteria.

 However, due to the lower recurrence rates achieved by the ultrasonically guided liver resection technique, this approach may be more suitable than liver resection combined with thermal ablation.

### 2.6. Liver Transplantation—A Future Opportunity?

Unfortunately, there are still many patients with multiple bilobar colorectal cancer liver metastases who are not amenable to complete resection by any of the above-mentioned therapeutic strategies. In such patients, the only chance of complete removal of the liver metastases is total hepatectomy followed by liver transplantation (Figure 1(g)). 

 This approach was used in the early period of liver transplantation, achieving 1- and 5-year overall survival rates of 62% and 18%, respectively [94]. 

Due to organ shortages, it was considered that the allocation of an organ to a patient with such a short life expectancy following the transplantation was not ethically acceptable.

Currently, unresectable CRLM are considered to be a contraindication to liver transplantation.

 However, the above-mentioned survival rates appear to be higher than those that can be achieved by palliative treatment, suggesting that even using the therapeutic options that were available thirty years ago, liver transplantation offered a higher survival benefit than the best palliative treatment that is currently available. Furthermore, due to recent progress in the fields of posttransplant immunosuppression and medical oncology and due to the more refined methods that are used in selecting patients to receive tailored therapies (based on reliable pathologic and biologic markers), improved survival rates could be achieved for selected patients who undergo liver transplantation.

 Moreover, the ethical issues could be challenged by the use of a living donor liver transplantation given that, in such instances, the willing donation is directed toward a certain patient and not to the community [95]. Meanwhile, it is also considered unethical to offer a marginal graft to a patient with a good chance of long-term survival following liver transplantation. Because the number of available marginal grafts has increased in recent years, it may be acceptable to allocate such organs in selected patients with CRLM, at least in the setting of controlled trials.

 Although the available data do not support liver transplantation as a routine procedure in patients with CRLM, we believe that a discussion of the current advances in this field and of the recently published results is worthwhile and should encourage debate on this issue. 

 By reviewing the largest series of patients undergoing liver transplantation for unresectable CRLM [96], it was revealed that 66% of patients with histologically negative lymph nodes were genetically positive for micrometastases when mutant allele-specific amplification (MASA) method was used to search for micrometastases in DNA from the regional lymph nodes of the primary colorectal cancer [97]. Those patients who were both genetically and histologically negative exhibited a significantly longer overall survival (*P* value = 0.011) than the other patients. Thus, Kappel et al. concluded that the genetic detection of micrometastases by MASA may be a powerful prognostic indicator for selecting patients with colorectal liver metastases who could benefit from liver transplantation [97].

 Over the previous two decades, certain further developments have emerged that may improve patient selection and the results of liver transplantation. For example, the advent of MDCT, gadolinium-enhanced MRI, and PET/CT scans has improved the detection of extrahepatic metastases, permitting the better selection of patients with colorectal cancer that had metastasized only to the liver. Recent studies have identified several biological parameters (such as the expression of p53, thymidylate synthase, Ki-67, K-ras, and human telomerase reverse transcriptase, as well as the type, density, and location of immune cells within the tumor) that may be more sensitive predictors of outcome in patients with CRLM than are the current histopathological methods that are used to stage colorectal cancer [98, 99]. Using a panel that incorporates these parameters, it may be possible to identify a highly selected group of patients who could greatly benefit from liver transplantation. Moreover, it has been hypothesized that the progress in posttransplant immunosuppressive therapy may decrease recurrence rates and improve the survival of patients who undergo liver transplantation for malignant disease, primarily due to the use of m-TOR inhibitors. Such immunosuppressive agents (sirolimus, temsirolimus) inhibit tumor growth and proliferation and exhibit antiangiogenic effects. These effects are in contrast to traditional immunosuppressive drugs, which appear to promote malignant cell proliferation [100, 101].

 Over the past several years, taking into account the better expertise of transplant surgeons and the above-mentioned progress in both the selection of patients with CRLM and in the efficacy of posttransplant immunosuppressive regimens, certain authors have argued that the outcome of selected patients who undergo liver transplantation for unresectable liver metastases from colorectal cancer may be significantly improved [102]. For these reasons, certain authors have proposed a rational revisitation of the concept of liver transplantation in such patients. Thus, a pilot study (SECA-study) that aims to assess the survival and quality of life in patients receiving pretransplant chemotherapy, liver transplantation for unresectable CRLM, and posttransplant Sirolimus-based immunosuppressive regimen began in Norway in November 2006. The preliminary data of this study reveal a 94% survival rate after a median 25 months of postoperative follow-up and an excellent quality of life [102]. However, only 40% of these patients are disease-free after a median follow-up period of 25 months.

 Favorable results were also recently reported in two patients with CRLM who were treated with liver resection followed by hepatic artery infusion chemotherapy and who underwent a liver transplantation for intraarterial chemotherapy-induced sclerosing cholangitis. The two patients were disease-free at 2 and 5 years following transplantation, respectively.

 Based on these disparate results, definitive conclusions cannot be drawn; however, due to ethical considerations (i.e., organ shortage), liver transplantation with grafts from brain-dead donors cannot be accepted until 5-year survival rates exceed 50% [102].

However, if the results that are achieved by liver transplantation will become significantly higher than those can be achieved by nonsurgical therapies, it may eventually be difficult, in the future, to defend the prohibition of living-donor liver transplantation or liver transplantation with marginal grafts in highly selected patients with unresectable CRLM. Such a position would be difficult given that ethical considerations would no longer be valid in such situations. 

## 3. Conclusions

Selected patients with initially unresectable CRLM may be rendered resectable following portal vein embolization or ligation, resulting in an important survival benefit or even a cure.

 “Two-stage” hepatectomies (with/without PVE/PVL) may be performed safely, achieving complete resection of liver metastases and long-term survival. 

The use of ultrasonographically guided hepatectomies decreases the requirement for major hepatectomies, portal vein occlusion and “two-stage” liver resections in patients with CRLM that are close to the hepatocaval confluence or in cases of multiple bilobar disease. This approach provides (1) an improved comfort and safety profile over “two-stage” liver resections and major hepatectomies following PVE/PVL and (2) a similar oncological benefit to other strategies.

Liver resection following conversion chemotherapy in previously unresectable patients may offer a considerable survival benefit. RFA could be combined with liver resection to increase the number of patients who are eligible for complete removal and ablation of CRLM.

In the future, highly selected patients with unresectable CRLM and favorable prognostic factors who receive liver transplantations with grafts from marginal donors or from living donors could achieve better survival rates than would be possible with palliative treatment. However, further studies and perioperative treatment improvements are required before this procedure achieves social acceptance.
